# Human Cardiac Stem Cells Isolated from Atrial Appendages Stably Express c-kit

**DOI:** 10.1371/journal.pone.0027719

**Published:** 2011-11-28

**Authors:** Jia-Qiang He, Duc Minh Vu, Greg Hunt, Atul Chugh, Aruni Bhatnagar, Roberto Bolli

**Affiliations:** Institute of Molecular Cardiology, University of Louisville, Louisville, Kentucky, United States of America; Brigham & Women's Hospital - Harvard Medical School, United States of America

## Abstract

The *in vivo* studies of myocardial infarct using c-kit^+^/Lin^−^ cardiac stem cells (CSCs) are still in the early stage with margin or no beneficial effects for cardiac function. One of the potential reasons may be related to the absence of fully understanding the properties of these cells both *in vitro* and *in vivo*. In the present study, we aimed to systematically examine how CSCs adapted to *in vitro* cell processes and whether there is any cell contamination after long-term culture. Human CSCs were enzymatically isolated from the atrial appendages of patients. The fixed tissue sections, freshly isolated or cultured CSCs were then used for identification of c-kit^+^/Lin^−^ cells, detection of cell contamination, or differentiation of cardiac lineages. By specific antibody staining, we demonstrated that tissue sections from atrial appendages contained less than 0.036% c-kit^+^/Lin^−^ cells. For the first time, we noted that without magnetic activated cell sorting (MACS), the percentages of c-kit^+^/Lin^−^ cells gradually increased up to ∼40% during continuously culture between passage 2 to 8, but could not exceed >80% unless c-kit MACS was carried out. The resulting c-kit^+^/Lin^−^ cells were negative for CD34, CD45, CD133, and Lin markers, but positive for KDR and CD31 in few patients after c-kit MACS. Lin depletion seemed unnecessary for enrichment of c-kit^+^/Lin^−^ cell population. Following induced differentiation, c-kit^+^/Lin^−^ CSCs demonstrated strong differentiation towards cardiomyocytes but less towards smooth and endothelial cells. We concluded that by using an enzymatic dissociation method, a large number, or higher percentage, of relative pure human CSCs with stable expression of c-kit^+^ could be obtained from atrial appendage specimens within ∼4 weeks following c-kit MACS without Lin depletion. This simple but cost-effective approach can be used to obtain enough numbers of stably-expressed c-kit^+^/Lin^−^ cells for clinical trials in repairing myocardial infarction.

## Introduction

It is a long-held belief that mammalian cardiomyocytes withdraw from the cell cycle during the perinatal period and that the mammalian heart is a terminal post-mitotic organ incapable of self-regeneration after myocardial injury. However, this paradigm has been challenged by the work of Beltrami and colleagues [1] who for the first time, discovered specialized cells within the heart tissue expressing stem cell markers (c-kit, Sca-1, and MDR1). These cells, termed adult cardiac stem cells (CSCs), are capable of fulfilling the criteria for stem cells including self-renewal, clonogenicity, and multipotency. To date, at least 5 different types of CSCs, including the c-kit^+^/Lin^−^ cells [1], [2]; the Sca-1^+^ cells [3], [4]; the Isl1^+^ cells [5], [6], the cardiac side population (Abcg2^+^/MDR^+^) [7], [8], and cardiosphere-derived stem cells (c-kit^+^/Sca-1^+^/Flk1^+^) [9]–[11], have been isolated and characterized from hearts by different laboratories [12]–[16]. There may be additional CSCs populations [17]–[19]. Although, the origin and the function of these cells remain unclear, different putative adult CSCs most likely represent different developmental and/or physiological stages of a unique CSC population in the adult mammalian heart [20].

The c-kit^+^/Lin^−^ cells represent one of the major CSC populations founded in the heart [21]. In both children and adults, these cells are present in highest number in the right atrial appendage [22], [23]. In vitro, the c-kit^+^/Lin^−^ CSCs show typical stem cell properties and pluripotency, and some in vivo studies have shown that transplantation of these cell improves cardiac function in animal models of myocardial infarct (MI). However, the results are variable. Some studies report marked improvement in function, whereas other report only marginal or non-significant effects on cardiac structural and/or function [13], [24]–[27]. It is likely that this variability stems from the lack of understanding of the biological properties of these cells before and after transplantation and how these cells could be reproducibly identified, isolated and transplanted. In particular, issues such as the specificity of c-kit as a CSC marker, contamination from other cell source, CSC lineage and the expression of cardiac lineage markers before and after differentiation remain controversial [16], [28]–[30].

The c-kit antigen is primarily expressed in hematopoietic stem cells, but its expression disappears in cells of hematopoietic lineage after differentiation, except for mast cells [31]–[33]. Therefore, it has been recently suggested that c-kit^+^ cardiac progenitor cells isolated from human heart tissue are actually mast cells [34]. Similarly, it is unclear whether or not the c-kit population is contaminated by other cells such as the cardiac fibroblasts, mast cells or hematopoietic lineage cells and whether the expression of c-kit remains stable after tissue processing and long-term cell culture. Data on the expression of lineage markers are even more conflicted. For instance, Anversa and colleagues reported that human c-kit^+^ cells (both cloned and uncloned CSCs), do not express all the cardiac lineage markers such as GATA4, Nkx2.5, MEF2c, α-sarcomeric actin (α-SA), α-smooth muscle actin (α-SMA), CD31, and KDR before differentiation as measured by flow cytometry; whereas 4–23% of the un-fractional cell population at P0 was found to be positive for these markers [12], [35], [36]. In their recent study, they reported a similar lack of expression of cardiac lineage markers, except for KDR, which was found to be expressed in ∼3% of c-kit^+^ population [36]. In contrast, Itzhaki-Alfia *et al* have reported that the un-fractioned adult human CSCs are positive for GATA4^+^ (60%) and cardiac α-SA^+^ (60%) even before c-kit purification [22]. In neonatal hearts and heart tissue from young children, however, the percentages of c-kit^+^ ranged from 5%–9% and only small percentage (1% to 3%) of c-kit^+^ cells were double positive for Nkx2.5^+^ [23]. Clearly, many aspects about these cells remain to be fully understood. Therefore, the present study was designed to systematically characterize the c-kit^+^/Lin^−^ CSCs isolated from adult human heart samples. We examined CSC markers adapted to post-isolation, expansion, purification, Lin depletion (Lin-dep); their vitro differentiation properties, and potential contamination by other cell types, such as cardiac fibroblasts and mast cells. The overall goal in this work was to provide essential but practical information required for optimizing the use of human CSCs for future clinical trials in MI repair.

## Materials and Methods

**Figure 1:**
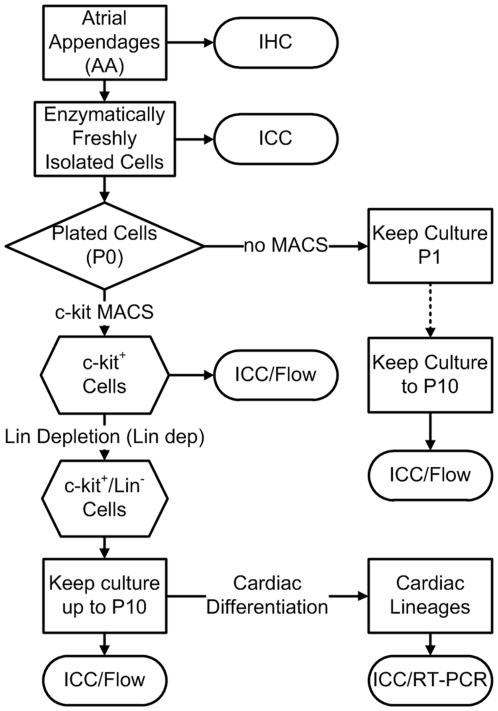
Experimental design. The samples of human atrial appendages (AA) were randomly sorted in 4 groups and used either for immunohistochemistry (IHC) or enzymatically dissociated into single cells for Immunocytochemistry (ICC) or Flow Cytometry (Flow). The cultured cells were then underwent c-kit magnetic activated cell sorting (MACS) and/or followed by Lineage depletion (Lin-dep.) or simply kept culturing without any purification and selection. The resulting c-kit^+^/Lin^−^ cardiac stem cells (CSCs) were used for cardiac differentiation and analyzed by Flow, ICC and RT-PCR.

Tissue samples of discarded atrial appendages from patients were randomly divided into 4 groups (Figure 1). Group 1 comprised of tissue sections for immunohistochemistry (IHC) to identify c-kit^+^ cell and cardiac α-SA or freshly isolated cells directly used for immunocytochemistry (ICC) with human c-kit specific antibody. Group 2 cells were continuously cultured at 37°C up to passage 10 (P10), but without any purification or enrichment steps using c-kit or Lineage (Lin) magnetic activated cell sorting (MACS). Group 3 cells were subjected to c-kit MACS and/or Lin-dep. In the last group, isolated cells went through single or double MACS procedures with c-kit and Lin antibodies and these cells were then used for cardiac differentiation. All isolated cells were passed every 4–5 days up to P10. The cells were collected on every other passage for ICC and flow cytometry using various antibodies anti c-kit, hematopoietic Lin markers, KDR, CD31, CD34, CD45, CD133, and cardiac lineage markers (such as Nkx2.5 and GATA4).

### Human atrial appendages

A total 30 atrial appendages specimens were collected from 30 patients during the routine procedure of open heart surgery from Jewish Hospital at University of Louisville. The specimens were from right atrial appendages and the size of samples varied from 50 to 500 mg. No information was able to be collected in terms of patients' age, sex, types of cardiac diseases, and properties of non-cardiac diseases, and other medical history of patients based on the approved protocol. A written consent agreement was obtained for collection of discarded atrial appendages according to a protocol approved by the Institutional Review Boards on human subject research at University of Louisville.

### Cell dissociation and culture

Enzymatic digestion was used to dissociate human CSCs from atrial appendages as described previously [12]. Briefly, after removing fat, the tissues were washed 3× with cold Ca^2+^/Mg^2+^-free PBS buffer. Thereafter, the samples were minced into small pieces (∼1 mm) and washed extensively in fresh cold PBS buffer. The chopped tissues were then transferred into 50 ml tube and incubated with collagenase II (30 U/ml) in 37°C shaker for 60 min. The loosened tissues were further mechanically dissociated by gently pipetting. The undigested tissue clumps were allowed to settle down by gravity on ice for 10 min followed by transferring the supernatant into another 15 ml tube. After centrifuge at 1,400 rpm for 5 min, the cell pellet was resuspended in fresh CSC culture medium consisting of Ham's F12 (Invitrogen, CA), 10% FBS (Hyclone, UT), 10 ng/ml human bFGF (PeproTech, NJ), 0.2 mM L-glutathione, and 0.005 U/ml human erythropoietin (both from Sigma, MO). The cells suspension was then plated in culture flask and cultured in 37°C incubator supplied with 95% air/5% CO_2_. Next day, the medium was entirely changed to remove all dead cells and cell debris. The adherent cells were continuously cultured with medium change every other day or split at 4–5 days with TrypLE Express (Invitrogen, CA). The subsequent analyses were done on the entire un-fractional population of cultured cells or after c-kit MACS and/or Lin-dep.

### Magnetic activated cells sorting (MACS)

The c-kit^+^/Lin^−^ CSCs were enriched using the c-kit MACS kit according to the manufacturer's instructions (Miltenyi Biotec, Germany). Briefly, total populations of adherent cells between P1 to P3 (dependent on cell density) were enzymatically dissociated into single cell suspension. The cells were collected and incubated with microbeads directly conjugated to mouse monoclonal anti-human c-kit antibody (clone AC126) at 4°C to 8°C for 20 min. The cells were then loaded into a MACS column which was placed in the magnetic field of MACS Separator (Miltenyi Biotec, Germany). The labeled c-kit^+^ cells were retained on the column and unlabeled cells were eluted. After column was removed from magnetic field, the magnetically retained c-kit^+^ cells were collected as positively selected cell fraction for further expansion and analysis with ICC, flow cytometry, and/or RT-PCR.

To exclude the potential contamination of Lin^+^ cells (such as T cells, B cells, NK cells, dendritic cells, monocytes, granulocytes, erythroid cells, and their committed precursors), a Lineage Cell Depletion Kit (Miltenyi Biotec, Germany) was used to purify c-kit^+^ cell populations. This kit is a biotin conjugated-monoclonal antibody cocktail consisting of CD2, CD3, CD11b, CD14, CD15, CD16, CD19, CD56, CD123 and CD235a antibodies. Similar to c-kit MACS, Lin-depletion required the cells to be incubated with the antibody cocktail at 4°C to 8°C for 15 min and then incubated with microbeads conjugated anti-biotin secondary antibody for another 15 min. The magnetically labeled Lin^+^ cells were depleted by retaining them on MACS column in magnetic field while unlabeled Lin^−^ cells passed through the column and were collected for further expansion and/or analysis.

### Flow Cytometry

Flow Cytometry was used to analyze the expression patterns of CSCs markers during cell culture. Briefly, after detaching adherent cells with TrypLE Express (Invitrogen), single cell suspension was filtered through 70 µm nylon mesh and then centrifuged and washed with 2× 5 ml PBS (Invitrogen). Cells were incubated on ice for 30 to 60 min (depending on individual antibody) with appropriate combination of fluorochrome conjugated-primary and/or secondary antibodies. The labeled cells were then washed 3× with PBS, fixed with 1% PFA for 10 min and resuspended in FACS buffer for flow analysis on LSRII Flow Cytometer (BD Bioscience) using FacsDave 6.0 software (BD Bioscience, CA) or FlowJo 7.5 (TreeStar, Ashland, USA). The following antibodies and dilutions used in Flow Cytometry and ICC (see below) included: (1) Unconjugated primary antibodies: rabbit IgG anti-c-kit; mouse IgG anti-GATA4 (both from Santa Cruz, CA); mouse IgG anti-Nkx2.5; mouse IgG anti-Fibroblast (both from Abcam, MA); mouse IgG anti-Tryptase (Chemicon, CA); mouse IgM anti-Cardiac α-SA; mouse IgG anti-α-SMA; rabbit IgG anti-KDR (all from Sigma, MO). (2). Fluorochrome directly conjugated primary antibodies: mouse IgG anti-CD31-APC; mouse IgG anti CD34-APC; mouse IgG anti CD45-APC; mouse IgG anti CD133-APC (all from eBioscience, CA); mouse IgG anti-Lin cocktail (CD2, CD3, CD14, CD16, CD19, CD24, CD56, CD68, CD123, CD235a-FITC (BD Bioscience, CA); (3). Secondary antibodies: donkey anti-rabbit IgG-FITC; donkey anti-mouse IgG-FITC (both from Invitrogen, OR); donkey anti-mouse IgM-TRITC (Jackson Immunoresearch, PA).

### Immunocytochemistry (ICC) and immunohistochemistry (IHC)

The expression of CSC markers were also analyzed by immunofluorescence techniques. Briefly, freshly isolated or detached cell suspension was subjected to live-cell staining or fixation with 4% PFA before primary antibody incubation, depending on the location of antigen, *i.e.*, an intra- or extra-cellular marker. Live-cells were incubated on ice with the primary antibody for 30–60 min and fixed-cells were incubated at room temperature for 20–45 min depending on individual antibody. In each case, PBS supplemented with 10% horse serum was used as blocking solution. The sample was incubated with an appropriate combination of fluorochrome conjugated and/or primary and secondary antibodies (see Flow Cytometry above). Cells were stained by DAPI (or Hoechst 33342 for live-cell) for identifying the nuclei. Samples were then centrifuged in Cytospin. Images were collected and analyzed using a LSM 510 META Confocal laser-scanning microscope and/or an Axiovision Fluorescence microscope (both Carl Zeiss, Germany). The percentage of positive cells of interest was estimated on the basis of total nuclei (∼300 to 600/per data set) counted from each sample slide of at least three patients.

IHC was used to determine the percentages of c-kit^+^ in tissue sections. Briefly, the fat was removed from atrial samples, and followed by washing 3× in cold PBS buffer to remove possible blood, and tissues were then fixed in 10% formalin solution for 24 h followed by 70% ethanol for another day at room temperature. The fixed tissue blocks were then embedded in paraffin and sectioned at a thickness of 4 µm. Negative controls were included each time by omitting primary antibody with a normal procedure. Following counter-staining with DAPI, slides were mounted with fluorescence anti-fade Vectashield mounting medium under cover slip. LSM 510 META Confocal and/or Axiovision fluorescence microscopes were used to acquire fluorescent images and percentage of c-kit^+^ was estimated based on total nuclei (∼3000) counted from three sections of each sample among at least three patients. The primary antibodies used were rabbit IgG anti-c-kit (Dako, CA) and mouse IgM anti-Cardiac α-SA (Sigma, MO); the secondary antibodies included donkey anti-rabbit IgG-FITC (Invitrogen, CA) and donkey anti-mouse IgM-TRITC (Jackson ImmunoResearch, PA).

### RT-PCR

Total RNA was extracted from cells using the PARIS Kit (Applied Biosystem, CA), and then treated with DNase to eliminate DNA contamination. The quality and quantity of total RNA was estimated with Nanodrop 2000c (Thermo Scientific, DE). RT-PCR was run with Qiagen Onestep RT-PCR kit according to manufacturer's instructions. For each measurement, 100 ng of total RNA was used as a template and RT-PCR was performed using primers (from IDT, Coralville, IA) listed below at concentration of 0.5 µM. Cycle conditions were: 50°C/30 min, 94°C/15 min, 94°C/30 s for denature, 58–60°C/30 s for annealing, and 72°C/30 s for extension with total cycles of 35. PCR products were run on 2% agarose/1×TBE gel stained with ethidium bromide for 30 min (Bio-Rad, CA). The following primers were used: *c-kit*: forward-TACTGAT TCACTGCATGGCTCCCA; reverse-ACAGAGTGCCTTAAGTGCAGGTGA. *GATA4*: forward -CGAATGACGGCATCTGTTTGCCAT; reverse-GCAGAACAGCAATGTGCAGAGGA. *ME F2c*:forward-TGGCTGCATGTTTGAATCAGGTGG; reverse-AGACCACCTGTGTTACCTGC ACTT. *KDR*: forward-AGAGGCTTTGTTTAGGACGTGGGT; reverse-TTGAACCTCCCGCA TTCAGTCTCA. *vWF*: forward-ACTCAGTGCATTGGTGAGGATGGA; reverse-TCGGACAC ACTCATTGATGAGGCA. *Cardiac troponin I (cTnI)*: forward-AATCAGACGCCGCTCCTC CAAC; reverse-TCTCCCACCTCCCGGTTTTCCTTC. *MLC2v*: forward-AGAGCTGGGCGGA GTGTGGAAT; reverse-TTTCACGTTCACTCGCCCAAGGG. *GAPDH*: forward-AAGGTCG GAGTCAACGGATTTGGT; reverse-AGTGATGGCATGGACTGTGGTCAT.

### In vitro differentiation

In vitro assays of cardiac differentiation were performed to examine three cardiac lineages, cardiomyocytes, smooth muscle, and endothelial cells, according to the modified method from previous published studies [22], [37]. Briefly, c-kit^+^/Lin^−^ CSCs were seeded in triplicates in either 6-well plates (for RT-PCR analysis) or T75 flasks (for ICC assay) and cultured for 1 to 2 days in human CSC medium. To induce cardiac differentiation, the culture medium was switched into differentiated medium consisting of DMEM (Invitrogen, CA), 10% FBS (Hyclone, NJ), and 10 µM 5-azacytidine (5-AZ, Sigma, MO) for 3 days. Cells were continuously cultured up to 35 days and the medium was changed every other day. Total RNA extracted from control (Day 0) and differentiated cells (Day 3, 7, 14, 21, 28 and 35) and used to analyze gene expression patterns of cardiac lineages (c-kit, GATA4, MEF2c, KDR, vWF, cTnI, and MLC2v) by RT-PCR with specific primers purchased from IDT (see above). Protein expressions was examined only on Day 0 before differentiation and Day 35 after differentiation by ICC using cardiac lineage-specific antibodies: rabbit IgG anti c-kit (Santa Cruz, CA), mouse IgG anti-CD31-APC (eBioscience, CA), mouse IgG anti-α-SMA, mouse IgM anti-cardiac α-SA (both from Sigma, MO), and rabbit IgG anti-cTnI (Chemicon, CA). The secondary antibodies consisted of: donkey anti-rabbit IgG-FITC; donkey anti-mouse IgM-TRITC or FITC (c-kit) or (α-SMA or α-SA) (all from Invitrogen, CA).

### Other materials

Human umbilical vein endothelial cells (HUVECs, CRL-1730), human fibroblasts (CRL-110), and human Jurkat cells (TIB-152) were obtained from ATCC (Manassas, VA). Human bone marrow cells were purchased from AllCells (Emeryville, CA). Total RNA of human carotid artery was bought from Agilent Technologies (Santa Clara, CA). Cells were grown according to manufacturer's product instructions. All chemicals used were from Sigma (MO) and medium from Invitrogen (CA), unless otherwise stated.

### Statistical analysis

The data are presented as mean ± SEM (standard error of mean). Student's t test with two-tailed distributions was used to compare two groups. One-way ANOVA followed by Bonferroni test was used to compare three and more groups. *p*<0.05 was considered significantly. Cells from at least 3 patients were used for each group.

## Results

### c-kit expression pattern in CSCs before and after c-kit MACS

**Figure 2:**
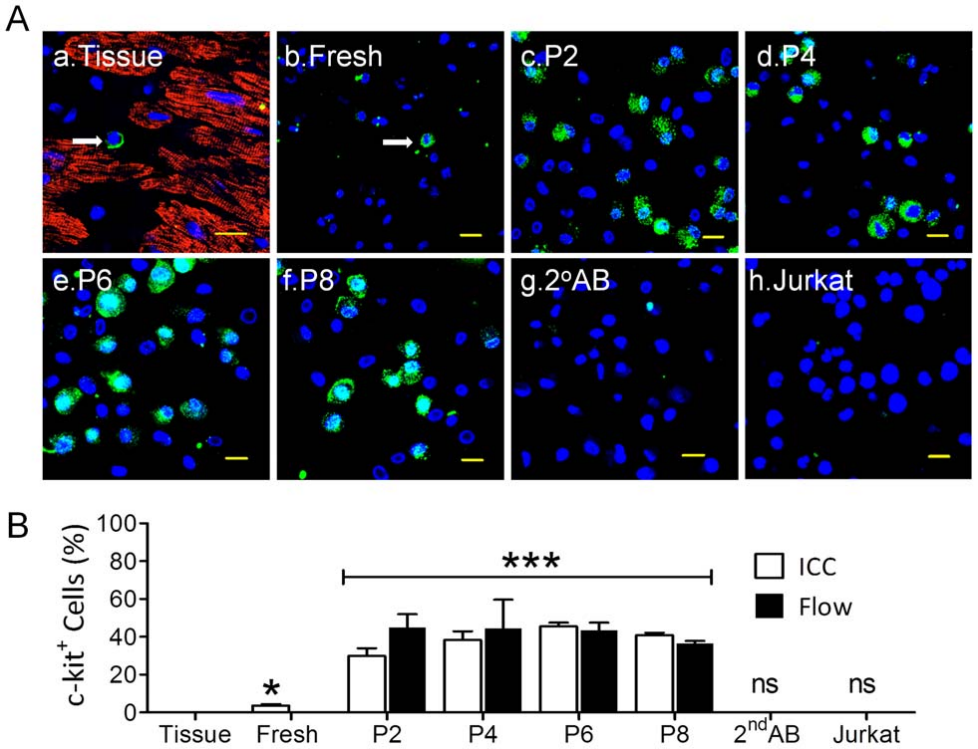
C-kit^+^ cells before purification. Panel A showed the representative images on tissue section (a) of atrial appendages or freshly isolated cells before (b) and after plating (c–f) at passage 2 (P2) to P8 from the same patient. C-kit^+^ cells were showed in green (FITC, see arrows in a and b, but all green cells in c–f) and nuclei were stained in blue (DAPI). The negative controls included secondary antibody (g. 2^nd^AB) alone and human Jurkat cells (h) stained with c-kit antibody. Scale bars = 20 µm. Panel B displayed the Mean±SEM data from ICC (open bar) and Flow Cytometry (filled bar). n = 4–9 patients. *p<0.05, ***: *p*<0.01, ns: no significance, all were compared to control (Tissue).

To ensure high quality and purity of the cells for future clinical trials, changes in c-kit expression were examined after isolation and/or long-term culture of human CSCs. As shown in Figure 2, c-kit^+^ cells were readily identified in section of the atrial appendages, however, only a small percentage (∼0.036%) of cells (counted by nuclear staining) in the atria displayed c-kit positivity (Figure 2A.a). Estimates of the percentage of c-kit^+^ cells in freshly-dissociated single cell population from atrial appendage indicated that about 3.6±0.82% cells were c-kit^+^ as measured by ICC (Figure 2A.b and 2B). The larger number of c-kit^+^ cells obtained in dissociated cells (*p*<0.05, n = 4 patients) may be due to more accurate measurement of the entire cell population than those with tissue sections. However, because of a large number of dead cells, cell debris and undissociated cells in the isolate, this estimate could potentially also result in an overestimation of c-kit^+^ cells. Nevertheless, both the techniques indicated that the abundance of c-kit^+^ cells in the atrial appendage is quite low.

To evaluate the stability of c-kit expression during long-term culture, we collected cells after every other passage to examine changes in c-kit expression by ICC and flow cytometry, separately. We noted that the percentages of c-kit^+^ cells were gradually increased from 3.6±0.82% in freshly isolated cells up to ∼40% between P2 to P8 before c-kit magnetic selection and purification (Figure 2). The average percentages of c-kit^+^ cells from P2 to P8 was significantly higher than tissues section and freshly isolated cells (*p*<0.01, n = 4 to 9 patients, Figure 2B). Because mature lymphocytes do not express c-kit [38], we used a cloned human Jurkat cell line (T lymphocyte) and 2^nd^ antibody as negative controls to exclude potential false positive. Our data showed that both control groups produced no c-kit^+^ staining (Figure 2A.g and h).

**Figure 3:**
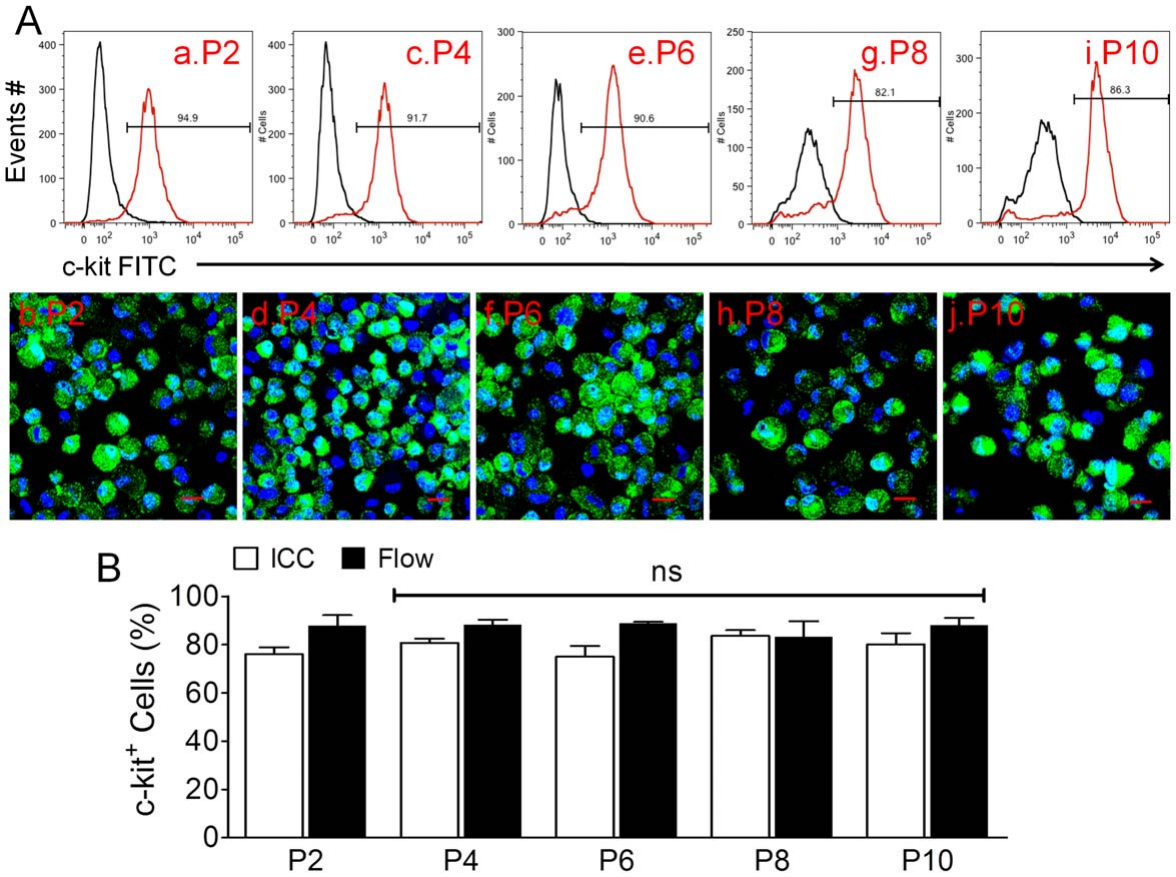
C-kit^+^ cells after purification. Panel A showed the representative data of Flow Cytometry (upper row), where c-kit-FITC was shown on x-axis, cell Events# was on y-axis, and the percentages of c-kit^+^ cells was in red histograms. The representative images of ICC from the same patient was displayed in middle row, where c-kit^+^ cells were showed in green (FITC) and nuclei were stained in blue (DAPI) at P2-P10 after c-kit MACS but without Lin-dep. Scale bar = 20 µm. Panel B displayed the Mean±SEM data from ICC (open bar) and Flow Cytometry (filled bar). The percentages of c-kit^+^ cells by ICC and Flow showed no statistical significant difference (ns) compared to P2. n = 3 patients.

To purify and enrich c-kit^+^ CSCs, cultured cells were enzymatically detached at passage 1 to 2 and c-kit MACS was performed through column with un-fractional single cell population after incubation with human c-kit antibody-conjugated magnetic beads (see Materials and Methods). As shown in Figure 3, the percentage of c-kit^+^ cells in the population increased dramatically up to ∼80%, as analyzed by flow cytometry (Figure 3A, upper row) and ICC (Figure 3A, lower row). On average, 79.1±3.2% and 87.2%±2.1% cells were identified to be c-kit^+^ by ICC and flow cytometry, respectively, between passage 2 and 10 (n = 3). There was no statistically significant difference between these passages, indicating that the c-kit expression was quite stable during long-term culture for up to 10 passages for a total of 40 to 50 days. In addition, karyotyping analysis of sorted CSCs was normal both at lower (P3) and high (P10) passages with 46 XY or YY based on total 60 cells from three patient samples (data not shown). Moreover, c-kit^+^ cells demonstrated the typical stem cell property of clonogenesis (data no shown). Collectively, these data indicate that the percentage of c-kit^+^ cells in intact heart section of atrial appendages is much lower than in freshly isolated cells and continuous culture of un-fractionated cells without any purification for c-kit. The population could be further enriched by c-kit MACS and that once isolated these cells stably express the c-kit antigen.

### Expression of hematopoietic markers in CSCs

Having established the abundance and the stability of c-kit^+^ cells in culture, we next examined whether the c-kit^+^ population isolated from the atrial appendage was contaminated by hematopoietic stem cells (which also express c-kit) or cells of hematopoietic lineage (which do not). For this, we examined several markers of hematopoietic cells. In addition to c-kit (CD117) hematopoietic stem cells express other hematopoietic markers, including CD31, CD34, CD38, CD45, CD59, CD90, CD105, CD106, CD133, CD164, CD184, CD201, and CD202 [39]. However, the mature hematopoietic cells (T/B lymphocytes, monocytes, granulocytes, and NK cells) express Lin markers, such as CD2, CD3, CD4, CD5, CD8, CD11b, CD13, CD14, CD16, CD18, CD19, CD20, CD24, CD33, CD56, CD61, CD66, CD71, CD 235, and Ter119 [39]. Therefore, to identify contaminating cells we measured these markers in the c-kit^+^ cell population.

**Figure 4:**
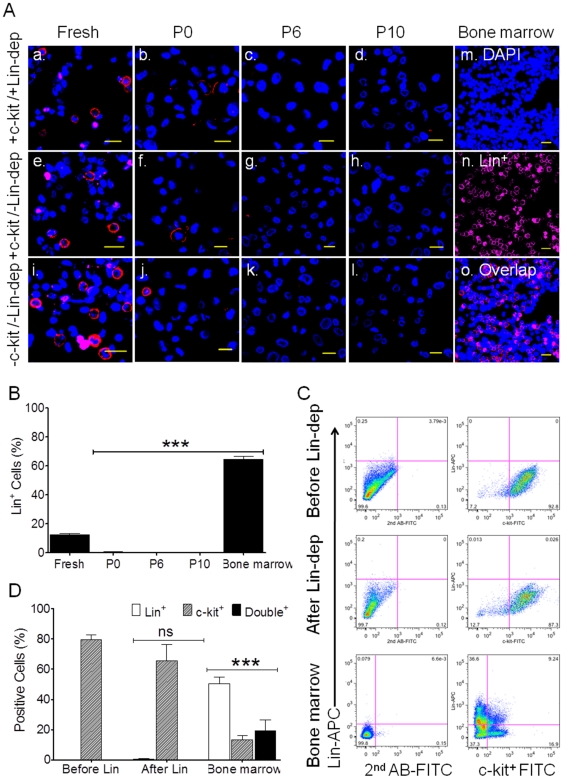
Lineage positive (Lin^+^) cells. Panel A showed the representative Confocal images of Lin^+^ antibody staining (red-APC) on freshly isolated cells as well as plated cells at P0, P6, and P10 with (+) and/or without (−) c-kit MACS and/or Lin-dep (Panel A, top row-with c-kit and Lin-dep; middle row with c-kit, but without Lin-dep; and bottom row without c-kit and Lin-dep). Cell nuclei were stained in blue-DAPI, and Scale bars = 20 µm. MACS were able to be performed at P1 to P2. Human bone marrow (m to o) was used as a positive control for Lin markers (red-APC). Panel B showed the Mean±SEM data from ICC. ***: *p*<0.001 (P0, P6, P10 and human bone marrow) compared with control group (Fresh isolated cells). Panel C displayed the representative data of Flow Cytometry with double staining of c-kit-FITC (x-axis) and Lin-APC (y-axis) in CSCs before (upper) and after (middle) Lin-dep. Human bone marrow was used as positive control (lower). Panel D showed the Mean±SEM) data of Flow Cytometry from three patients. ***: *p*<0.001 and ns: no significance; both were compared with control level (Before Lin-dep).

We found that without any selection procedures, freshly isolated cells from the atrial appendage contained ∼10% of Lin^+^ cells (Figure 4A.a, e, I and 4B) as identified by ICC using a mixed Lin antibody cocktail consisting of CD2, CD3, CD14, CD16, CD19, CD20 and CD56. To exclude the possible contamination from hematopoietic stem cells the following three sets of experiments (n = 3 patients for each set) were performed in various combination of c-kit MACS and Lin-dep showed in Figure 4: (a) Freshly isolated cells were subjected to c-kit MACS followed by Lin-dep between P1 to P2 (top row). After a two-step MACS procedure, barely any Lin^+^ cells were seen (Figure 4A.c, d and 4B); (b) Freshly isolated cells were subjected to c-kit MACS, but no Lin-dep, in which Lin^+^ cells disappeared afterwards (middle row, Figure 4A.g., f, 4B); (c) Freshly isolated cells were continuously cultured without c-kit MACS and Lin-dep, where Lin+ cells also disappeared (bottom row, Figure 4A.k, l and 4B). In all three groups, the average percentage of Lin^+^ cells was 12.3±0.9% in freshly isolated cells (n = 3 patients), however, these cells were undetectable after one or two passages whether or not Lin-dep step was applied. However, the percentages of c-kit^+^ cells remained high with or without Lin-dep (data not shown), similar to the levels shown in Figure 2.

To confirm these results, the cells were treated with c-kit (-FITC) v.s. Lin (-APC) specific antibodies, and antibody binding was detected by flow cytometry. As showed in Figure 4C and 4D, Lin^+^ cells were not detectable after c-kit MACS whether or not Lin-dep was used. Although the percentages of c-kit^+^/Lin^−^ cells were slightly reduced after c-kit MACS, from 79.5±3.1% (before Lin) to 65.6±10.8%, the difference did not reach statistical significance (Figure 4D, ns, n = 3 patients). In control experiments with human bone marrow cells using two different measurements (ICC vs Flow), we obtained comparable percentages of Lin^+^ cells −64.7±2.0% by ICC (Figure 4B) vs 50.5±4.4% by flow cytometry (Figure 4D). A small population 13.4±2.5% of cells was c-kit^+^ (Figure 4D; n = 3 in each group).

**Figure 5:**
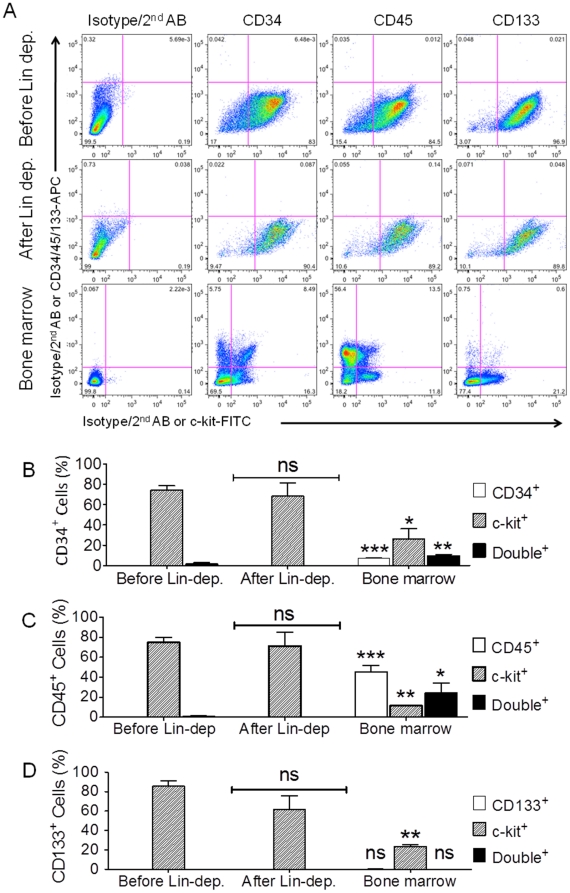
Expression of hematopoietic markers. Panel A displayed the representative data of Flow Cytometry with double staining of c-kit-FITC (x-axis) vs CD34, CD45, or CD133 (all-APC in y-axis) in c-kit^+^ cell population before (top row) and after (middle row) Lin-dep. Human bone marrow (bottom row) was used as positive control. Panel B, C, and D represent the Mean±SEM data of CD34, CD45, and CD133, respectively. Human bone marrow cells were used as positive control in each group. *: *p*<0.05, **: *p*<:0.01, ***: *p*<0.001; ns: no significance. Each data was compared with the corresponding control levels (Before Lin-dep.).

To assess contamination by hematopoietic stem cells, the expression of hematopoietic stem cells markers (CD34, CD45 and CD133) was analyzed in the c-kit^+^ cell population. Although the percentages of c-kit^+^ cells remained high at 74.3±4.3% and 68.5±13.3% (CD34), at 74.8±4.8% and 71.4±13.5% (CD45), and at 85.6±5.6% and 61.6±14.2% (CD133) before and after Lin-dep (n = 3 patients in each group), respectively, there was no detectable signals of CD34^+^, CD45^+^ and CD133^+^ cells in c-kit^+^ cells whether or not Lin-dep was used (Figure 5). Using human bone marrow cells as positive control, we found that 20.3±6.6% (ranging from 11±0.85% in CD45, 23.7±1.6% in CD133, to 26.1±10.4% in CD34 group, respectively) were c-kit^+^, whereas the abundance of CD34^+^ cells was 4.5±1.8%, CD45^+^ cells was 45.9±10.5%, and CD133^+^ cells was 0.5±0.2% (Figure 5B, 5C, and 5D, n = 3 patients). Collectively, these data indicate that single-step purification by c-kit MACS is sufficient to produce a highly enriched c-kit cell population and that the inclusion of an additional step of Lin-dep does not lead to an increase in the percentage of c-kit^+^ cells in long-term culture up to 10 passages. The resultant c-kit^+^ cell population did not express Lin markers or typical hematopoietic stem cell markers (like CD34, CD45, or CD133), indicating that cell population after c-kit MACS is not contaminated by hematopoietic stem cells or mature leukocytes of hematopoietic origin.

### Contamination by fibroblasts and mast cells

**Figure 6:**
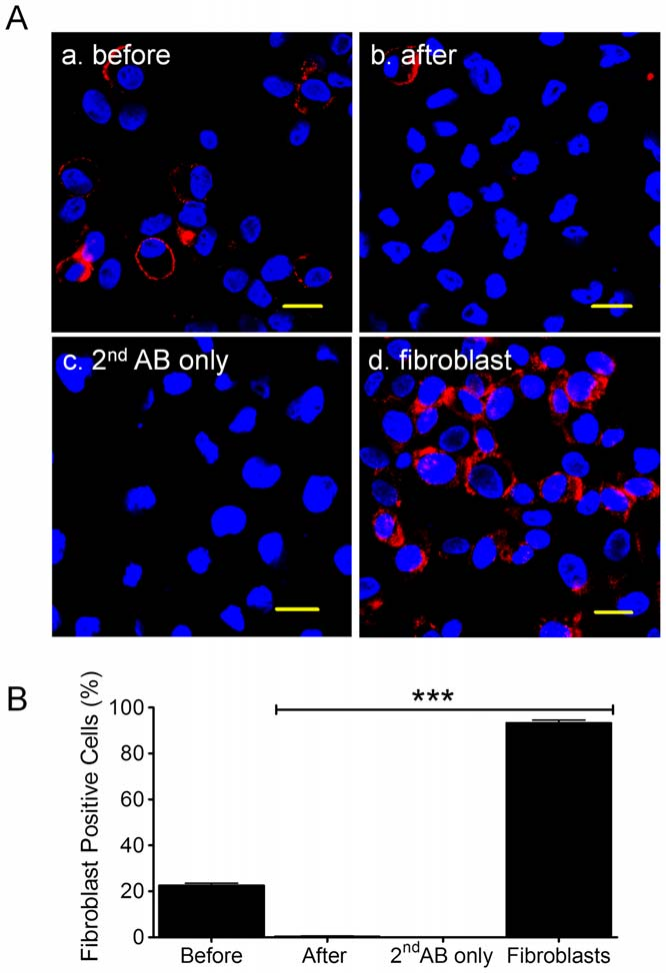
Fibroblast specific antibody staining. Panel A showed the representative images of fibroblast antibody (Fibroblast Surface Protein in red-APC) staining on human CSCs before (a) and after (b) c-kit MACS. The 2^nd^ antibody-APC (c, 2^nd^ AB only) and human fibroblast cell line (d) were used as positive and negative controls, respectively. Cell nuclei were stained in blue (DAPI). Scale bars = 20 µm. Panel B showed the Mean±SEM data of ICC staining from three patients (n = 3 patients). ***: *p*<0.001 compared with control (Before c-kit MACS).

Previous studies have shown that cardiac fibroblasts represent a heterogeneous population in the heart with regard to their phenotype, specific functions, and cellular origins [40]. To exclude the possibility that isolated cells from atrial appendages are fibroblasts or contaminated with fibroblasts, we measured the abundance of fibroblasts in the c-kit^+^ cell population using a fibroblast-specific antibody raised against “Fibroblast Surface Protein” (FSP) [41]–[44]. We found that a small proportion of un-fractional cells isolated from atrial appendages were fibroblast positive cells (22.7±0.9%, n = 3 patients); however, these fibroblasts were entirely removed by c-kit MACS procedure (Figure 6). As a positive control, we examined human fibroblasts, which showed that 93.3±1.2% of the population was FSP^+^, whereas a negative control using 2^nd^ antibody failed to generate a positive signal for fibroblasts (Figure 6).

**Figure 7:**
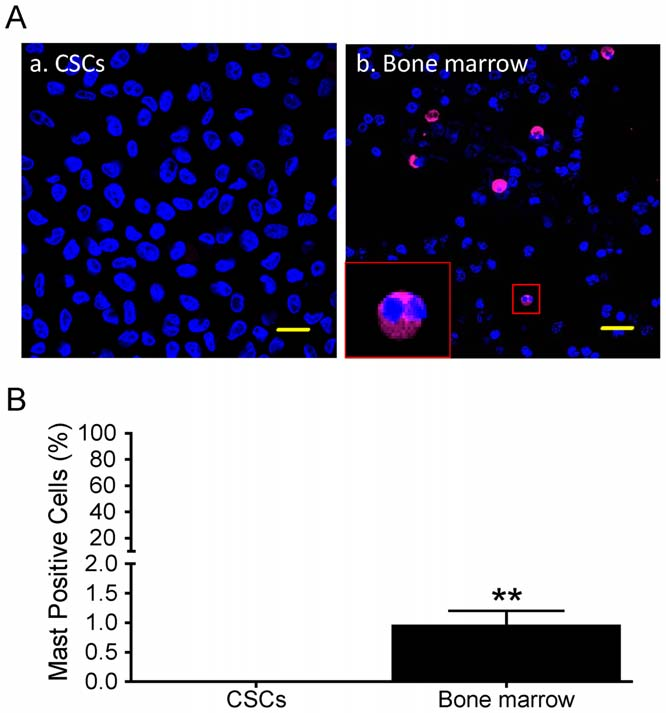
Mast cell specific antibody staining. Panel A demonstrated the representative Confocal images of mast cell specific antibody staining (Tryptase, red-APC) on c-kit^+^ CSCs (a). Human bone marrow (b) was used as positive control; the inset in b was an enlarged image of a mast positive cell. Nuclei were stained in blue (DAPI). Scale bars = 20 µm. Panel B showed the Mean±SEM of ICC data from three patients (n = 3 patients). **: *p*<0.001 compared to human CSCs.

Although the c-kit antigen has been widely used to identify stems cells in the heart [13], this antigen is also expressed by cardiac mast cells [45]. Thus, in principle, it is possible that some of the stem cell recognized in the heart may be mast cells. To exclude this possibility, we used a well-documented mast cell-specific antibody, Tryptase [23], [33], [34], [46] to examine mast cell contamination in the sorted c-kit^+^ cell population. However, as shown in Figure 7, we did not observe any tryptase^+^ mast cells in c-kit^+^ cells isolated from three different patients (n = 3). In contrast, a small percentage (0.95±0.3%, n = 3) of human bone marrow cells displayed positive staining by the mast cell antibody (Figure 7). These data clearly showed that the c-kit^+^ cell population isolated from atrial appendages was not contaminated by fibroblast and c-kit reactivity of these cells could not be attributed to the presence of cardiac mast cells.

### Cardiac lineage differentiation

**Figure 8:**
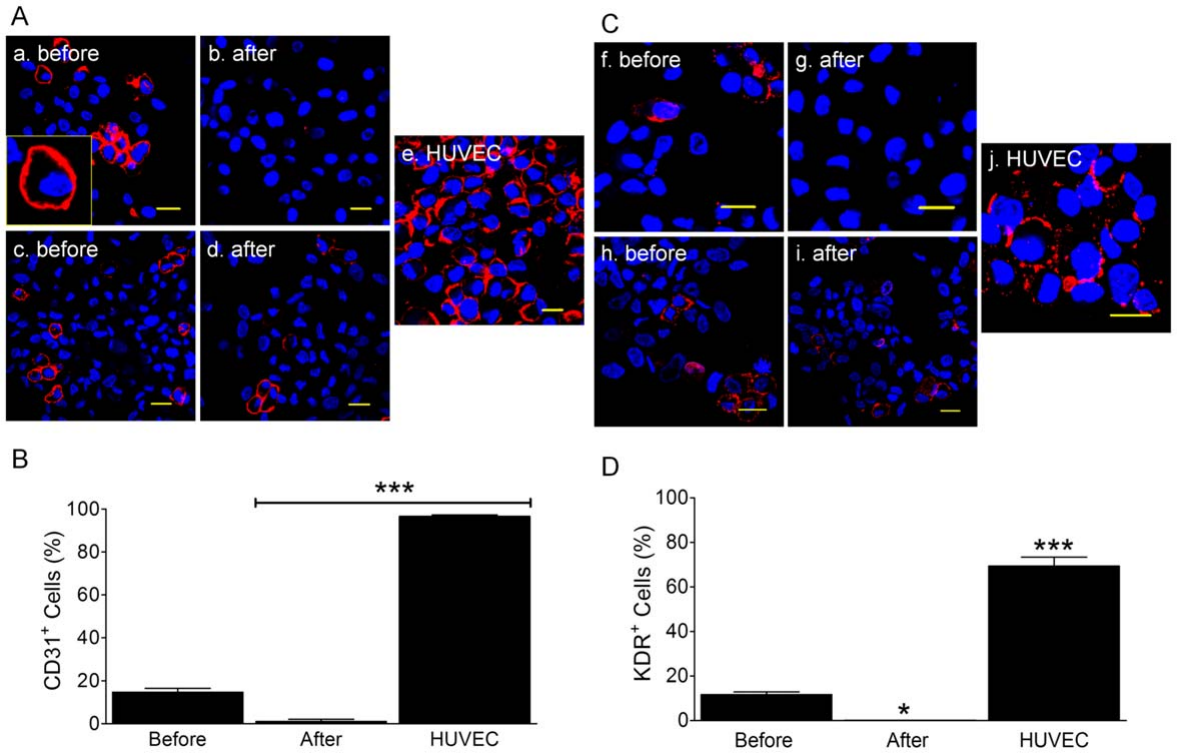
Endothelial specific antibody staining. Panel A showed the representative images of CD31 antibody staining before (a and c) and after (b and d) c-kit MACS in two different subtypes of cell populations in patients. Majority patients (Group 1) did not displayed CD31^+^ cells after c-kit MACS (a and b), but few patients (Group 2, n = 2) still demonstrated the reproduced CD31^+^ cells after c-kit MACS (c and d). In all images, CD31^+^ cells were labeled in red (APC) and nuclei were stained in blue (DAPI). HUVECs (e) were used as positive control. Scale bars = 20 µm. Panel B only showed the Mean±SEM of CD31 antibody staining in Group 1 (n = 5 patients). ***: *p*<0.001 compared to control (Before c-kit MACS). Panel C confirmed the above results with different endothelial specific antibody (KDR). The percentages of KDR^+^ cells before (f, h) and after (g, i) c-kit MACS in two groups (f and g in Group 1, h and i in Group 2) were similar to those in CD31 experiments mentioned above. In the images, KDR^+^ cells were labeled in red (APC) and nuclei were stained in blue (DAPI). HUVECs (j) were used as positive control. Scale bars = 20 µm. Panel D only showed the Mean±SEM of KDR antibody staining of Group 1. n = 3 patients. *: *p*<, ***: *p*<0.001 compared to control (Before c-kit MACS).

The CSCs are believed to be the pre-committed cardiac-resident stem or progenitor cells and therefore, they should carry typical stem cell and/or cardiac markers (like c-kit and GATA4 etc). These cells also have the capability to differentiate into three cardiac lineages both in vitro and in vivo under certain condition. Therefore, we examined changes in markers of cardiomyocytes (MEF2C, GATA4, Nkx2.5, α-SA, cTnI, and MLC2v), smooth muscle cells (α-SMA), and endothelial markers (CD31, KDR, and vWF) before and after differentiation of the c-kit^+^ cell population. Before induced differentiation, the unsorted cell population from a majority of patients contained 17±2.5% CD31^+^ cells as measured by ICC (Figure 8A.a, and 8B, n = 5 patients) and 21.7±2.6% CD31^+^ cells as measured by flow cytometry (n = 3 patients, data not shown). These “contaminating” CD31^+^ cells could be removed by c-kit MACS (Figure 8A.b). However in two cases (2 out of 7; Figure 8A.c and d, n = 2), unsorted cells demonstrated similar percentages (15% and 21%) of CD31^+^ cells before c-kit MACS, but interestingly, but 18–20% CD31^+^ cells were still present after c-kit MACS (data not shown), indicating a unique subtype of c-kit^+^/CD31^+^ among c-kit^+^ cell population. Moreover, in a majority of patients (5 out of 7) 11.7±1.2% of the cells identified by ICC and 19.1±1.2% of the cells identified by flow cytometry (n = 3 patients, data not shown) were KDR^+^ before c-kit MACS (Figure 8C.f. and 8D, n = 5 patients). Like the CD31^+^ cells, the KDR^+^ cells could also be removed by c-kit MACS (Figure 8C.f and g). However, in two patients, the KDR^+^ cells in c-kit^+^ population were not able to be removed before (13% and 17%, also see Figure 8C.h) or after (18% and 16%, also see Figure 8C.i) c-kit MACS, indicating the presence of a c-kit^+^/KDR^+^ sub-population of cells (Figure 8C. h and i). Dual staining of c-kit^+^ cells with CD31 and KDR antibodies indicated that they co-express these antigens in various combination, suggesting existence of c-kit^+^, c-kit^+^/CD31^+^/KDR^+^, or CD31^+^/KDR^+^ (data not shown). When HUVECs were used as a positive control, we observed high percentages of CD31^+^ (96.7±0.7% by ICC and 97.3±0.7% by flow, Figure 8B) as well as KDR^+^ cells (69.3±4.1% by ICC and 68.3±2.0% by flow; Figure 8D) in the cell population. From these data we conclude that the c-kit^+^ cells contain a distinct population of CD31^+^/KDR^+^ cells. Because these cells were observed in only a sub-set of patients, their presence in the heart may be related to the medical history of these patients, their disease condition or other clinical factors. However, for now, their potential role remains unclear.

**Figure 9:**
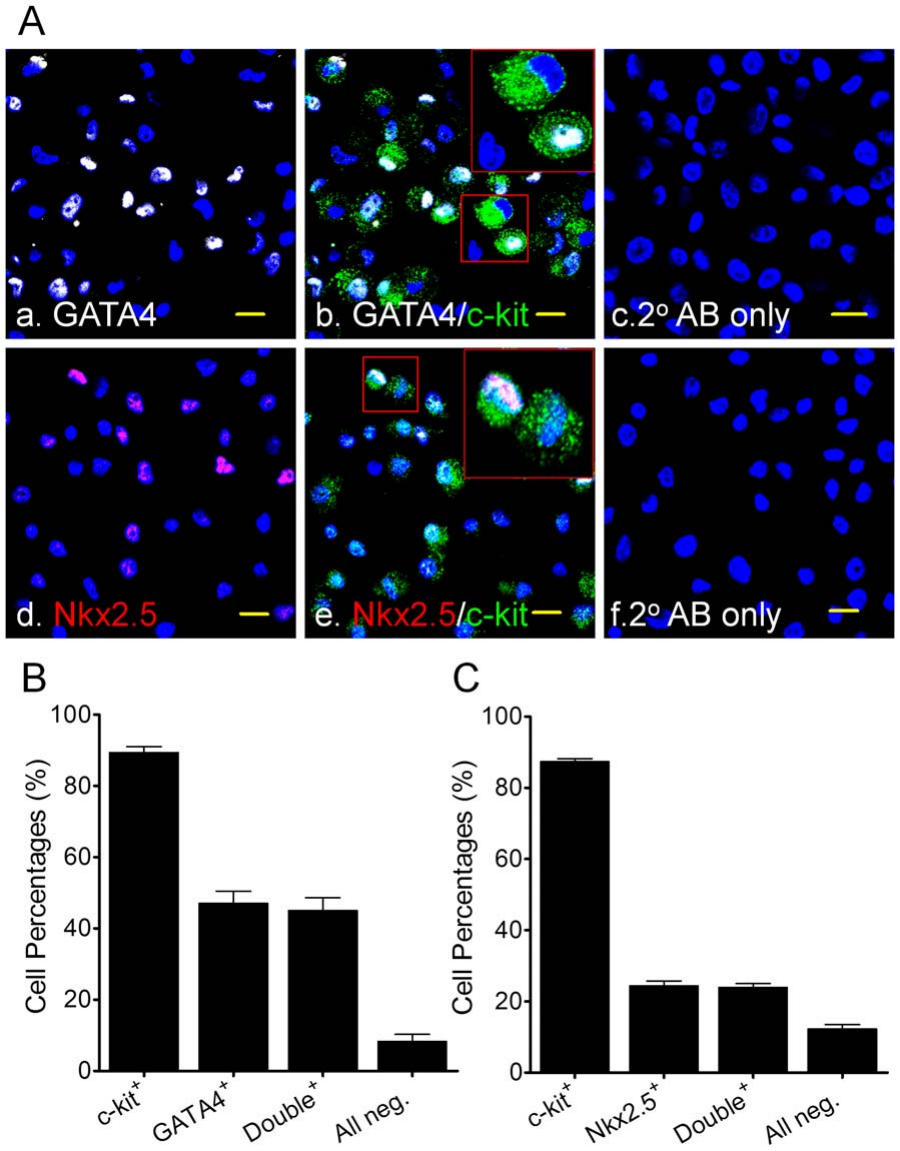
Early cardiac transcription factor staining. Panel A showed the representative Confocal images of GATA4 (upper row, white) and Nkx2.5 (lower row, red) antibody staining on human c-kit^+^ CSCs. Insets in b and e showed the enlarged overlap images of GATA4^+^/c-kit^+^ and Nkx2.5^+^/c-kit^+^ cells, respectively. The secondary antibody (2° AB) conjugated with FITC or APC were used as negative control. c-kit was stained in green (FITC) and nuclei were stained in blue (DAPI). Scale bars = 20 µm. Panel B and C showed the Mean±SEM of GATA4 and Nkx2.5 ICC data from three patients, respectively, where single positive (c-kit^+^) and double positive (c-kit^+^/GATA4^+^, left panel; c-kit^+^/Nkx2.5^+^, right panel) cells were counted and percentages were plotted.

**Figure 10:**
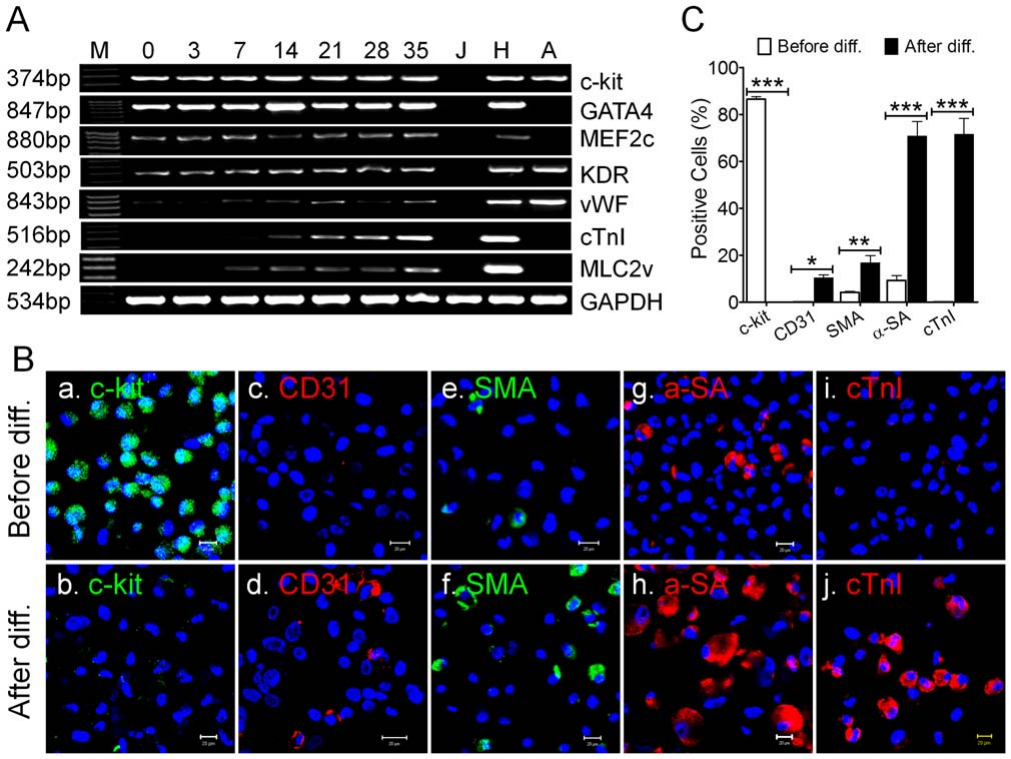
Cardiac lineage differentiation induced by 5-azacytidine. Panel A displayed the representative RT-PCR data of cardiac lineage genes (cardiac genes: *GATA4*, *MEF2c*, *cTnI*, and *MLC2v*; endothelial genes: *KDR* and *vWF*) before (Day 0) and after differentiation (Day 3, 7, 14, 21, 28, and 35). Stem cell marker (*c-kit*) and *GAPDH* were also included as a control. Total RNA of human Jurkat cell (J), human Heart (H) and human carotid artery (A) were used as negative and positive controls, respectively. DNA molecular marker (M) was shown the first column as M (bp). Panel B showed the representative Confocal images of c-kit (green-FITC), CD31 (red-APC), α-SMA (green-FITC), α-SA (red-APC), and cTnI (red-APC) before (upper) and after (lower) differentiation. Nuclei were stained in blue (DAPI). Scale bars = 20 µm. Panel C showed the MEAN±SEM of quantitative ICC data from three patients (n = 3 patients). *: *p*<0.05, **: *p*<:0.01, ***: *p*<0.001. Each data was compared with its control levels (before differentiation).

Although the sorted c-kit^+^ cells expressed several early cardiac-specific transcription factors (GATA4 & Nkx2.5, Figure 9), the expression of late cardiac lineage markers (α-SA, cTnI, MLC2v, VWF, CD31, & α-SMA) was low or undetectable both at gene and protein levels (Figure 10). Specifically, RT-PCR data clearly showed specific bands for GATA4 & MEF2c in c-kit^+^ cells, but no expression of late cardiac genes (cTnI and MLC2v) was observed (Figure 10). By ICC analysis, we confirmed that >80% of sorted c-kit^+^ cell population expressed c-kit^+^ antigen (also see Figure 3), among which there were about 47±3.5% GATA4^+^, 24.3±1.5% Nkx2.5^+^, 9.3%±2.2% α-SA^+^, 4.1±0.4% α-SMA^+^ cells. No cTnI^+^ cells were detected (Figure 9 and Figure 10, n = 3 patients for each group). We found that the GATA4^+^ or Nkx2.5^+^ cells were always c-kit positive, but c-kit^+^ cells were not always GATA4^+^/Nkx2.5^+^ positive (Figure 9).

Measurements of α-SMA by ICC demonstrated low levels of expression (4.1±0.4%, n = 3 patients) in c-kit^+^ populations before differentiation (Figure 10). The low abundance of this antigen may reflect the double positive cells (c-kit^+^/α-SMA^+^) found in tissue section of the human heart [12]. No positive staining was found when the secondary antibody alone was used as a negative control (data not shown). The presence of α-SMA^+^ cells in c-kit^+^/Lin^−^ CSCs suggests that some of these cells are pre-committed to a cardiac lineage in situ before ex vivo differentiation.

To induce cardiac differentiation, c-kit^+^/Lin^−^ CSCs were treated with 10 µM 5-azacytidine (5-AZ) for three days [22], [37]. Total RNA was extracted for RT-PCR analysis before (Day 0) and after differentiation on Day 3, 7, 14, 21, 28 and 35. As shown in Figure 10A, c-kit (stem cell marker), GATA4, MEF2C (both are cardiac markers) and KDR (endothelial marker) mRNA were expressed both before and after differentiation. However, after differentiation, the expression of vWF (endothelia marker), cTnI and MLC2v (late cardiac markers) was gradually increased. The mRNAs appeared on Day 7 and were continuously increased thereafter. No significant differences were observed in the levels of these mRNA between Day 28 and Day 35. In these experiments, total RNA extracted from human heart (H) and human carotid artery (A) was used as positive control, whereas Jurkat cells (J) were used as negative control (Figure 10). No detectable signal was observed with Jurkat cells (J), although all cardiac lineage markers and endothelial markers were robustly detected in samples from the heart (H) and artery (A), suggesting true expression of the genes of interest. To further examine expression levels of cardiac proteins following differentiation, we performed ICC using cardiac lineage-specific antibodies anti-CD31, α-SMA, α-SA, and cTnI. As it shown in Figure 10B and 10C, >85% CSCs expressed c-kit antigen before differentiation, however, the c-kit signal disappeared on Day 35 after differentiation although c-kit mRNA could still be detected (Figure 10A). In contrast, in we did not find CD31^+^ (endothelial marker) and cTnI^+^ (cardiac marker) cells in the c-kit^+^ population before differentiation by ICC, but a small percentage of α-SMA^+^ (4.1±0.4%) and cardiac α-SA^+^ (9.3±2.0%) was detected (Figure 10, n = 3 patients). Following cardiac differentiation, these markers were dramatically increased on Day 35. The CD31^+^ cells increased from 0 to 10.3±1.2%, α-SMA^+^ cells from 4.1±0.4% to 16.7±3.5%, α-SA^+^ cells from 9.3±2.0% to 70.7±6.2%, and cTnI^+^ cells from 0% to 71.4±6.9% (n = 3 patients in each group, *p*<0.05 or *p*<0.001, Figure 10). These data suggest that the pre-committed c-kit^+^/Lin^−^ CSCs are capable of differentiating into three cardiac lineages after induction by 5-AZ. Although a majority of the c-kit^+^ (about 70%) tend to become cardiomyocytes, some cells (about 30%) differentiate into smooth muscle and endothelial cells.

## Discussion

The results of the current study show that both tissue sections and cells freshly isolated from human atrial appendages specimens contain a small population of c-kit^+^/Lin^−^ cells. Without further purification, a gradual increase in the percentage of c-kit^+^ cells was observed, which increased to 40%. The percentage of c-kit^+^ cells did not increase further upon Lin-depletion following c-kit MACS. Nevertheless, c-kit expression was stable during long-term culture up to passage 10. Moreover after c-kit MACS, c-kit^+^/Lin^−^ cells were negative for hematopoietic stem cells (CD34, CD45, CD133) and Lin markers, however, in a few patients cells positive for endothelial marker (KDR/CD31) were observed. Lin-dep procedure seemed unnecessary for further enrichment of c-kit^+^ cells. No contamination from fibroblasts and mast cells was found. Importantly, c-kit^+^ CSCs could differentiate into three cardiac lineages. A major of these cells were transformed into cardiomyocytes, while a small population differentiated into smooth muscle and endothelial cells. Although several laboratories have published studies describing the isolation and characterization of human c-kit^+^/Lin^−^ CSCs [9], [12], [22], [23], [47] the stability of the stem cell characteristics of the population, contamination from hematopoietic stem cells or differentiation into cells of cardiovascular lineage has not been clearly established and therefore remain controversial [34], [45], [48].

### Stable expression of c-kit in CSCs

Recent evidence supports the view that the adult heart contains a discrete population of self-renewing stem cells [1], [36], [49], [50]. Using different stem cell markers and isolation methods, at least five different types of CSCs (c-kit^+^/Lin^−^; Sca-1^+^; Isl1^+^, cardiac side population/Abcg2^+^/MDR^+^ cells, and cardiospheres/c-kit^+^/Sca-1^+^/Flk1^+^) have been described [13], [21]. Among these, the c-kit^+^/Lin^−^ population has been most widely studied. These cells demonstrate typical stem cell properties of self-renewing, clonogenesis, and multipotency with the ability to differentiate into cardiomyocytes, smooth muscle cells, and endothelial cells *in vitro* [1]. Although in some *in vivo* studies c-kit^+^/Lin^−^ cells have shown beneficial results against cardiac remodeling after MI, in others no effect or only marginally significant effects were observed [13], [51]–[54]. This inconsistency may be related to variations in procedures used for cell isolation and transplantation and the lack of a consistent protocol for preserving the stemness of these cells and minimizing contamination by other cells.

In normal human heart, c-kit^+^ CSCs reside in discrete stem cell niches, but their overall abundance in the heart is rather low. Although some investigators have attempted to quantify the percentage of c-kit^+^ cells in whole heart [1], it is difficult to obtain accurate estimates based on quantification of heart section or freshly isolated cells. Quantification of c-kit^+^ cells by counting every single cell (including c-kit^+^ cells) in the entire human heart is tedious and often not feasible. On the other hand, counting these cells in fresh cell isolates is confounded by the presence of a large amount of dead cells, cell debris and clumps, making it difficult to get an accurate estimate of the abundance of the c-kit^+^ population either by flow cytometry or immunestaining. Nevertheless, our histological analysis of the atrial appendage indicated that the abundance of these cells in the human hearts is similar to that reported for the rat heart [12]. Because the right atrium is believed to be the highest levels of these cells [22], [23], it is likely the percentage of c-kit^+^ in other parts of heart might be even lower. Thus, expansion of c-kit^+^ cells from samples of the ventricle or the septum is likely to be more difficult than from atrial tissue and would require a longer time to allow the cells to grow to numbers required for clinical transplantation.

The impact of long-term cell culture on the enrichment of c-kit^+^ cells before purification has not been systemically assessed either. The published data are variable. Itzhaki-Alfia *et al* found that the dissociated human right-atrium generated 24±2.5% of c-kit^+^ before c-kit^+^ cell purification [22]., whereas Messina *et al* have reported that in cardiospheres-derived stem cells of murine heart there were ∼30% c-kit^+^ cells at Day 6 vs 10% at Day 0 after platting [9]; where Davis *et al* demonstrated that in rat heart, there were ∼12% c-kit^+^ cells on Day 21 vs 5% at Day 7 after culture [55]. We found that c-kit^+^ cells were gradually increased from very lower level in freshly isolated preparation up to 10% to 40% within two passage (about ten days) before c-kit MACS procedure. During the long-term culture (about 40–50 days), the percentage of c-kit^+^ cells remained stable at about 30–40% without any purification processes. The underlying mechanisms for this increase in c-kit^+^ cells remain unknown, but the expansion of these cells probably depends upon natural selection exerted by culture medium, which may favor or facilitate the proliferation of c-kit^+^ CSCs over other cell types [9], [56]–[58]. However despite of such selection, the abundance of c-kit^+^ cells did not exceed 80% unless c-kit MACS step was applied. Following this procedure, the purified c-kit^+^ cell population were be able to maintain its c-kit expression at a high level for more than month thus providing a uniform and stable population appropriate for clinical use.

### Contaminations of other cells types

Following the initial study reporting c-kit^+^ CSCs in the rat heart [1], several investigators have identified CSCs from hearts of various species, including mice [9], [59], rats [60], dogs [61], pigs [62], and humans [12], [36]. These studies were focused mainly on c-kit^+^/Lin^−^ cells populations, with little consideration of contamination from other cell types such as the mast cells, which are also c-kit^+^. In addition to CSCs, the c-kit antigen also expressed on other tissues including hematopoietic stem cells from the bone marrow [63], [64], cardiac mast cells [33], [34], melanocytes [65], interstitial cells of Cajal, prostate stem cells [66], and others [67]. Indeed within the population of cells isolated from the atrial appendage using enzymatic dissociation, we did find small percents of contaminating cell population expressing Lin, the fibroblast antigen, KDR or CD31. Although most of contaminated cells could be excluded using c-kit MACS, but in many cases the purity of c-kit^+^ could not reach 100%, suggesting that other un-identified cell types, not identified by current approaches and cellular markers, may be present in the preparation. Nevertheless, we confirmed that a majority (>80%) of sorted CSCs were c-kit^+^ and the rest of contaminated cells (if any) are not mast cell, fibroblasts, hematopoietic stem cells, and/or lineage cells. These data are consistent with other published studies [1], [12], [13], [23], [36], [47]. However, Zhou *et al* have recently reported that 85% to 100% c-kit^+^ cells isolated from human left ventricle were mast cells that displayed a weak or moderate signal for CD45, but were strongly positive for mast-cell markers such as Tryptase, Toluidine blue and Thionine [34]. In contrast, we found that c-kit^+^ cells were negative for mast cell marker even before c-kit purification and that these cells were also CD45 negative (as confirmed by flow cytometry and ICC using cells from at least three different patients). This disparity may be due to several reasons including the use of different experimental conditions, methods, patient, and especially tissue used to isolate the cells. For instance, Zhou used biopsy from left ventricle tissues v.s. the atrial appendages used in the present study. Another potential reason for undetectable mast cells in the present study may be due to non-adherent property of mast cells, which were probably washed away when medium was changed next day. It is known that the optimal methods are critical for the successful identification of true cardiac mast cells [68], hence the use of culture conditions described here should preclude contamination from mast cells and facilitate high yield recovery of a uniform population of c-kit^+^ CSCs.

### Cardiac differentiation potential

A variety of CSCs isolated from humans and experimental models [13] have been differentiated into cardiovascular lineages using different culture condition, including co-culture with mature or neonatal cardiomyocytes [5], [8], [69], [70], induction with 5-azacytidine [4], Oxytocin [3], or Dexamethasone [1], [12], [36]. Although these c-kit^+^/Lin^−^ CSCs have been widely studied, attempts to differentiate these cells in vitro have led to inconsistent results. It has been previously reported that in intact tissues sections from human hearts, the percentage of c-kit^+^ cells in right atrial tissue decreases from 8.9±0.4% in neonates to 6.4±0.2% to 3.2±0.1% in children from 2 to 13 years. Resident CSCs have been found to be most abundant in hearts of neonates [23]. However in adult human heart, the number of c-kit^+^ cells in even lower 0.01% [12], which was comparable to our estimates presented here. Because c-kit^+^/Lin^−^ cells are pre-committed resident CSCs, it is likely that they exist in heart in various stages of development and therefore they express cardiac lineage markers. Published data support this hypothesis. In human heart during early development c-kit^+^ and Nkx2.5^+^ were co-expressed in some CSCs with relative lower levels that range from 2.9±0.1% in neonates, 1.5±0.1% in infants to 1.0±0.1% in children 2 to 13 years [23]. In adult hearts, however, several different populations of stem cells committed to the cardiac lineage have been identified. These include (a) pre-committed cardiomyocytes co-expressing c-kit^+^/Nkx2.5^+^, c-kit^+^/α-SA^+^, c-kit^+^/α-SA^+^/Nkx2.5^+^, c-kit^+^/MEF2c^+^, c-kit^+^/α-SA^+^/MEF2c^+^ in CSC niches of tissue sections of rat [1], mouse [71], and human hearts [12], [72]; (b) Pre-committed endothelial progenitors co-expressing c-kit^+^/vWF^+^, c-kit^+^/KDR^+^, c-kit^+^/vWF^+^/KDR^+^; and (c) Pre-committed smooth muscle cells co-expressing c-kit^+^/KDR^+^/α-SMA^+^ in vascular niches of human hearts [1], [12], [72]. However, due to a variety of technical reasons, the actual number of each type of pre-committed CSCs within the entire heart has not been reported [12], although their abundance has been presumed to be low. Nevertheless, previous published data as well as our present data suggests that several types of pre-committed CSCs at various differentiation stages exist in adult hearts and therefore it is possible to obtain or identify these cells after dissociation.

In vitro single cell preparations of cardiac cells provide an alternative approach for identifying pre-committed CSCs with specific antibodies including anti-c-kit and/or other cardiac lineage markers or by using the more sensitive RT-PCR technique to detect cardiac genes expression. Previous studies from several laboratories have reported variable levels of c-kit^+^ expressions in cultured cells before purification and differentiation. Beltrami *et al* [1] found that the percentage of c-kit^+^ cell was 7–10% in cells isolated from the rat heart; but in cells isolated from the adult human right atrium, nearly 20 to 40% of the cells [12] [22] were found to be c-kit^+^. This estimate is similar to our finding that 10–40% of the cells in isolate were c-kit^+^ before purification and differentiation. Characterization of early myogenic transcription factors of CSCs isolated from human heart has shown that 60±6% [22] or 59±14% [12] of the cells were GATA4^+^ whereas 60±11% [22] of the cells were also positive for the late cardiac marker (α-SA). In agreement with these results we found that nearly 50% of cells were positive for both c-kit and GATA4. However, the abundance of c-kit^+^ cells expressing Nkx2.5, α-SMA, and α-SA was low. Based on cardiac lineage makers and their capacity for differentiation, D'Amario *et al* have suggested that c-kit^+^ CSCs could be classified into two groups - the “myogenic CSCs” (mCSCs) and the “vasculogenic CSCs” (vCSCs) [36]. This classification was based on their data showing that most of the mCSCs became cardiomyocytes, whereas the vCSCs differentiate primarily into endothelial and smooth muscle cells [36]. However, in their study, before differentiation, the c-kit^+^ cell population was negative for the cardiovascular markers - GATA4, Nkx2.5, MEF2c, α-SA, α-SMA, CD31, and TGF-β-1R and only a small percent of cells (3.3±1.1%) were positive for KDR, as determined by flow cytometry [12], [36], [72]. This is inconsistent with the previous study using the same technique [12] or the results obtained by other investigators [22], [23] including our data presented here. Hence, the extent of differentiation of human CSCs in situ or before in vitro differentiation remains unclear.

Cardiac lineage differentiation of c-kit^+^/Lin^−^ cells have been studied in various laboratories using either chemical reagents (such as Dexamethasone) or co-culture with neonatal or matured cardiomyocytes [13]. It has been reported that treatment with Dexamethasone results in the appearance of cardiac markers GATA4, Nkx2.5, MEF2c, and cardiac α-SA) in ∼50% of mCSCs, whereas ∼10–15% of the cells show smooth muscle and endothelial markers (GATA6, α-SMA, CD31, and vWF) after differentiation [36]. On the other hand, ∼40%–50% vCSCs became positive for smooth muscle and endothelial markers (GATA6, α-SMA, CD31, and vWF), and small percents of them (∼10–15%) showed positive for cardiac markers (GATA4, Nkx2.5, MEF2c, and α-SA) after differentiation [36]. In our study upon treatment with 5-AZ [37], we observed that a majority (∼70%) of c-kit^+^ cells differentiated into cardiomyocytes, but small percent (∼30%) of c-kit^+^ cells became smooth muscle and endothelial cells, demonstrating more efficient cardiomyocyte differentiation has been reported than before [36].

In conclusion, we have systemically and quantitatively characterized c-kit^+^/Lin^−^ CSCs isolated from human atrial appendages. We have developed appropriate procedures and protocols for maintaining purified c-kit^+^ cells. The cells maintained in culture stably expressed high percentage of c-kit antigen without contamination by other cells types except for in a few cases by CD31^+^ and KDR^+^ cells. Upon induction of differentiation, CSCs were able to become cardiomyocytes, smooth muscle and endothelial cells. We conclude that by using the simplified enzymatic dissociation method, a large number, but higher percent of relative pure c-kit^+^/Lin^−^ CSCs could be obtained from atrial appendages specimens within ∼4 weeks following c-kit MACS without Lin-dep. This simple, but cost-effective approach could be used to obtain sufficient numbers of stable c-kit^+^ cells for transplantation into patients with heart failure and myocardial infarct.
