# Correction: Human Cardiac Stem Cells Isolated from Atrial Appendages Stably Express c-kit

**DOI:** 10.1371/annotation/e67c06d9-a4b3-4d6b-837c-456bb87999b0

**Published:** 2012-01-19

**Authors:** Jia-Qiang He, Duc Minh Vu, Greg Hunt, Atul Chugh, Aruni Bhatnagar, Roberto Bolli

There was an error in the Acknowledgments. The spelling of the name "Dr. Yuro Guo" is incorrect. The correct spelling is "Dr. Yiru Guo". 

