# Correction: Human Cardiac Stem Cells Isolated from Atrial Appendages Stably Express c-kit

**DOI:** 10.1371/annotation/4849ca99-22e5-4082-a8e0-5c675168ffc2

**Published:** 2012-01-19

**Authors:** Jia-Qiang He, Duc Minh Vu, Greg Hunt, Atul Chugh, Aruni Bhatnagar, Roberto Bolli

There was an error in the text in the "Cell dissociation and culture" section of Materials and Methods. The correct text is: "The chopped tissues were then transferred into 50 ml tube and incubated with collagenase II (5mg/ml) in 37°C shaker for 60 min."

