# Correction: Human Cardiac Stem Cells Isolated from Atrial Appendages Stably Express c-kit

**DOI:** 10.1371/annotation/c27e7ce5-699d-4df6-9fea-03e84852f1de

**Published:** 2012-01-19

**Authors:** Jia-Qiang He, Duc Minh Vu, Greg Hunt, Atul Chugh, Aruni Bhatnagar, Roberto Bolli

There was an error in the text in the third paragraph in Results. The correct text is, "In addition, karyotyping analysis of sorted CSCs was normal both at lower (P3) and high (P10) passages with 46 XY or XX based on total 60 cells from three patient samples (data not shown)."

